# Engineered biosynthesis of plant heteroyohimbine and corynantheine alkaloids in *Saccharomyces cerevisiae*

**DOI:** 10.1093/jimb/kuad047

**Published:** 2023-12-22

**Authors:** Moriel J Dror, Joshua Misa, Danielle A Yee, Angela M Chu, Rachel K Yu, Bradley B Chan, Lauren S Aoyama, Anjali P Chaparala, Sarah E O'Connor, Yi Tang

**Affiliations:** Department of Chemical and Biomolecular Engineering, University of California, Los Angeles, Los Angeles, CA 90095, USA; Department of Bioengineering, University of California, Los Angeles, Los Angeles, CA 90095, USA; Department of Chemical and Biomolecular Engineering, University of California, Los Angeles, Los Angeles, CA 90095, USA; Department of Chemical and Biomolecular Engineering, University of California, Los Angeles, Los Angeles, CA 90095, USA; Stanford Genome Technology Center, Stanford University, Stanford, CA 94305, USA; Department of Chemical and Biomolecular Engineering, University of California, Los Angeles, Los Angeles, CA 90095, USA; Department of Molecular Cell and Developmental Biology, University of California, Los Angeles, Los Angeles, CA 90095, USA; Department of Chemical and Biomolecular Engineering, University of California, Los Angeles, Los Angeles, CA 90095, USA; Department of Bioengineering, University of California, Los Angeles, Los Angeles, CA 90095, USA; Department of Chemical and Biomolecular Engineering, University of California, Los Angeles, Los Angeles, CA 90095, USA; Department of Chemical and Biomolecular Engineering, University of California, Los Angeles, Los Angeles, CA 90095, USA; Department of Natural Product Biosynthesis, Max Planck Institute for Chemical Ecology, Jena 07745, Germany; Department of Chemical and Biomolecular Engineering, University of California, Los Angeles, Los Angeles, CA 90095, USA; Department of Chemistry and Biochemistry, University of California, Los Angeles, Los Angeles, CA 90095, USA

**Keywords:** Metabolic engineering, Monoterpene indole alkaloids, Strictosidine, Microbial production, Saccharomyces cerevisiae

## Abstract

Monoterpene indole alkaloids (MIAs) are a class of natural products comprised of thousands of structurally unique bioactive compounds with significant therapeutic values. Due to difficulties associated with isolation from native plant species and organic synthesis of these structurally complex molecules, microbial production of MIAs using engineered hosts are highly desired. In this work, we report the engineering of fully integrated *Saccharomyces cerevisiae* strains that allow *de novo* access to strictosidine, the universal precursor to thousands of MIAs at 30–40 mg/L. The optimization efforts were based on a previously reported yeast strain that is engineered to produce high titers of the monoterpene precursor geraniol through compartmentalization of mevalonate pathway in the mitochondria. Our approaches here included the use of CRISPR-dCas9 interference to identify mitochondria diphosphate transporters that negatively impact the titer of the monoterpene, followed by genetic inactivation; the overexpression of transcriptional regulators that increase cellular respiration and mitochondria biogenesis. Strain construction included the strategic integration of genes encoding both MIA biosynthetic and accessory enzymes into the genome under a variety of constitutive and inducible promoters. Following successful *de novo* production of strictosidine, complex alkaloids belonging to heteroyohimbine and corynantheine families were reconstituted in the host with introduction of additional downstream enzymes. We demonstrate that the serpentine/alstonine pair can be produced at ∼5 mg/L titer, while corynantheidine, the precursor to mitragynine can be produced at ∼1 mg/L titer. Feeding of halogenated tryptamine led to the biosynthesis of analogs of alkaloids in both families. Collectively, our yeast strain represents an excellent starting point to further engineer biosynthetic bottlenecks in this pathway and to access additional MIAs and analogs through microbial fermentation.

**One Sentence Summary:**

An *Saccharomyces cerevisiae*-based microbial platform was developed for the biosynthesis of monoterpene indole alkaloids, including the universal precursor strictosidine and further modified heteroyohimbine and corynantheidine alkaloids.

## Graphical Abstract

**Graphical Abstract:**
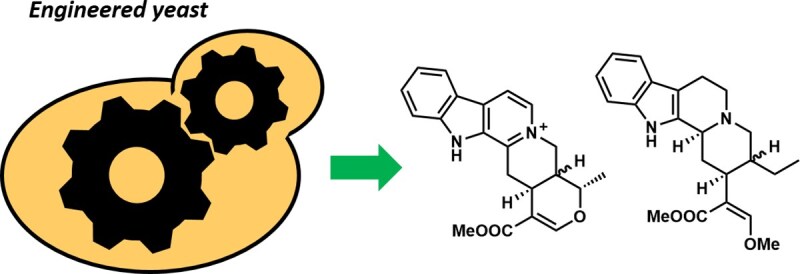

## Introduction

Monoterpenes indole alkaloids (MIAs) span a wide range of bioactivities, many with remarkable therapeutic values mitigating diseases such as cancer, Alzheimer's, and malaria (Chaturvedi et al., 2022; Heijden et al., 2005; Mohammed et al., 2021; Salim et al., 2023). These structurally complex compounds are isolated from a variety of plant species, such as vinblastine and vincristine from Madagascar Periwinkle (*Catharanthus roseus*) (Beckers & Mahboobi, 2003; Heijden et al., 2005), camptothecin from the Happy Tree (*Camptotheca acuminata*) (Lorence & Nessler, 2004; Wall et al., 1966) and mitragynine from kratom (*Mitragyna speciosa*) (Kruegel et al., 2016; Takayama, 2004). Members of this family have been listed in the WHO List of Essential Medicine due to their clinical relevance. However, the low abundance of certain MIAs from the native producers has resulted in high costs of the compounds for therapeutic applications (Bucar et al., 2013; Leonard et al., 2009). The difficulties in chemical synthesis of these compounds due to their structural complexity have further complicated sourcing of the MIAs (Sakamoto et al., 2020). As a result, there have been significant efforts in recent years in the engineered biosynthesis of MIAs from microbial or model plant hosts (Brown et al., 2015; Kim et al., 2023; Liu et al., 2022; Zhang et al., 2022). In particular, Baker's yeast (*Saccharomyces cerevisiae*, referred to as yeast from hereon), due to its generally regarded as safe status, abundance of synthetic biology tools, and an unparalleled track record for refactoring plant biosynthetic pathways, has emerged to be a top candidate for general MIA biosynthesis (Galanie et al., 2015; Li et al., 2018; Luo et al., 2019; Ro et al., 2006).

**Fig. 1:**
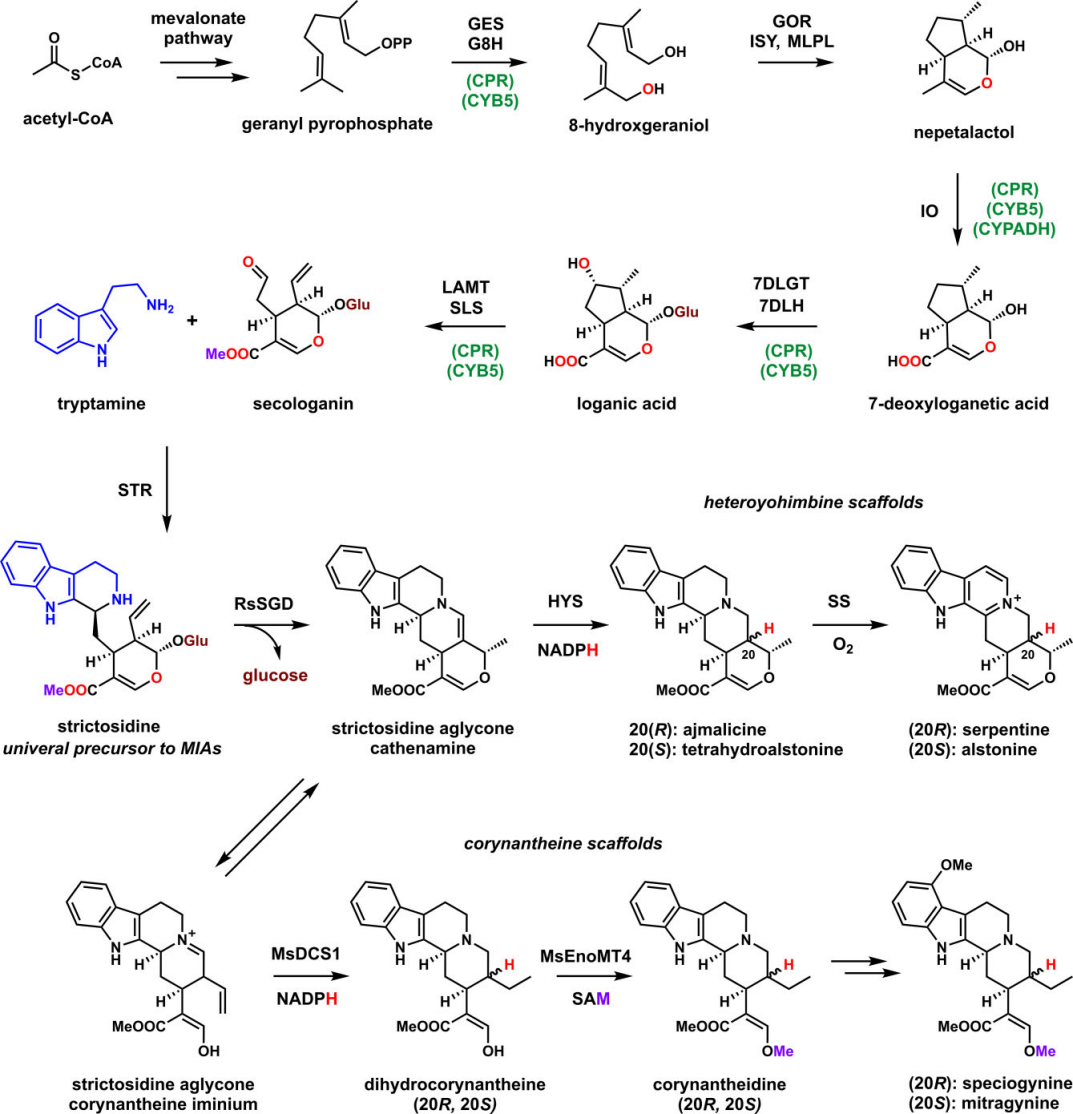
Biosynthetic pathway of heteroyohimbine and corynantheidine alkaloids. The biosynthetic enzymes are indicated for each step. Those indicated above or to the left of the arrow are pathway specific enzymes while those below or to the right of the arrow, in parentheses, are accessory enzymes. GES: geraniol synthase; G8H: geraniol 8-hydroxylase; GOR: 8-hydroxygeraniol oxidoreductase; ISY: iridoid cyclase; MLPL: major latex protein-like; IO: iridoid oxidase; 7DLGT: 7-deoxyloganetic acid transferase; 7DLH: 7-deoxyloganic acid hydroxylase; LAMT: loganic acid O-methyltransferase; SLS: secologanin synthase; STR: strictosidine synthase; RsSGD: *Rauvolfia serpentina* strictosidine β-D-glucosidase; HYS: heteroyohimbine synthase; SS: serpentine synthase; MsDCS1: *M. speciosa* medium-chain alcohol dehydrogenase 1; and MsEnoMT4: *M. speciosa* enol *O*-methyltransferase. Cofactors are indicated for steps after STR.

The biosynthetic pathways to heteroyohimbine and corynantheine families of MIAs are shown in Fig. 1. Both pathways, along with all other MIAs including the iboga families that vinblastine belongs to, are derived from the universal MIA precursor strictosidine (Caputi et al., 2018; Stavrinides et al., 2016). The enzymes required to synthesize strictosidine from the monoterpene precursor geranyl diphosphate (GPP) have been completely elucidated and reconstituted in heterologous hosts such as yeast (Collu et al., 2002; Geu-Flores et al., 2012; Uesato et al., 1986). In plants, the next step in MIA biosynthesis is the hydrolysis of glucose by strictosidine β-D-glucosidase (SGD) to reveal the reactive strictosidine aglycon (O'Connor & Maresh, 2006; Stöckigt et al., 1977). This aglycon can equilibrate in various forms in plants, including the enamine cathenamine and the corynantheine iminium. Reduction of cathenamine by reductases such as heteroyohimbine synthase (HYS) can give the mixed stereoisomers ajmalicine and tetrahydroalstonine (Stavrinides et al., 2016), which can be further oxidized by serpentine synthase (SS) or alstonine synthase to give serpentine and alstonine, respectively (Yamamoto et al., 2021). These compounds have potent antipsychotic and anxiolytic effects (Boğa et al., 2019; Elisabetsky & Costa-Campos, 2006). In *Mitragyna speciosa* that produce corynantheine compounds such as mitragynine, the iminium intermediate is first reduced by a medium-chain alcohol dehydrogenase (MsDCS1) to give a diastereomeric pair of dihydrocorynantheine, followed by enol methylation by MsEnoMT4 to give corynantheidine (Schotte et al., 2023). The 20*S* isomer of corynantheidine is further processed by undiscovered enzymes to give mitragynine, which is the most abundant alkaloid in kratom and has recently garnered interest for its therapeutic potential as an alternative to opioids (Flores-Bocanegra et al., 2020; Takayama, 2004). With such detailed biochemical information in hand, recent synthetic biology efforts by O'Connor, Keasling, Lian, Qu, and our groups have resulted in a number of MIAs produced from yeast and other model organisms, either *de novo* from the complete reconstitution of the target pathways (Brown et al., 2015; Liu et al., 2022; Zhang et al., 2022), or through feeding of strategically selected precursors (Kim et al., 2023; Misa et al., 2022). These efforts demonstrated feasibility of microbial production of complex MIAs, while also opening the door to opportunities for strain optimization toward titer improvement.

Our lab recently reported the biosynthesis of ∼50 mg/L of strictosidine using engineered yeast expressing enzymes in the strictosidine biosynthetic pathway (Misa et al., 2022). The engineered yeast platform contained several features: (1) the integration of accessory enzymes, cytochrome P450 reductase (CPR), CYB5, and CYPADH, into the yeast genome; (2) optimal combinations of inducible and constitutive promoters in controlling expression of pathway enzymes. This strategy alleviated the significant growth defects when all biosynthesis enzymes are expressed from constitutive promoters; and (3) use of single copy vector to express P450 enzymes such as iridoid oxidase (IO), 7-deoxyloganic acid hydroxylase (7DLH), and secologanin synthase (SLS), which presumably alleviates the ER-stress associated with expressing multiple foreign P450s. Starting from the precursor geraniol, scaled-up cultures of the yeast strain led to the first isolation and full nuclear magnetic resonance (NMR) characterization of strictosidine produced from yeast (Misa et al., 2022). However, notable drawbacks were also observed in this strain despite the high titer of strictosidine and strain robustness. First, a fully integrated strain is desired to circumvent plasmid stability issues (Mikkelsen et al., 2012). Second, the feeding of geraniol, which is cheap and abundant, resulted in significant accumulation of shunt products due to crosstalk of the pathway and endogenous yeast redox enzymes as a result of high concentration of geraniol (Billingsley et al., 2019). Therefore, a fully integrated *de novo* platform starting from a high GPP-producing yeast strain, followed by combining key features of the geraniol-feeding host described above, should overcome these shortcomings. In this work, we present engineering efforts to afford a yeast strain that can produce comparable levels of *de novo* strictosidine to our geraniol-fed strain. The resulting yeast strain was then demonstrated to be a suitable host for the biosynthesis of heteroyohimbine and corynantheine alkaloids, as well as fluorinated analogs using 7-fluorotryptamine.

## Results and Discussion

### General Strategy for De Novo Production of MIAs in Yeast

We previously engineered a yeast strain S25 for improved GPP production (Table 1) (Yee et al., 2019). The main feature of S25 was overexpression of the mevalonate pathway in the yeast mitochondria. This strategy took advantage of the high flux of acetyl-CoA in the mitochondria and avoided conversion of GPP to farnesyl pyrophosphate (FPP) in the cytosol by the essential FPP synthase (FPPS) (Campbell et al., 2016). With this strategy, we achieved ∼25 mg/L of geraniol biosynthesis in test tube cultures, and ∼227 mg/L titer of 8-hydroxygeraniol in fed-batch fermentation (when G8H is expressed). When downstream enzymes needed to form nepetalactol, which included 8-hydroxygeraniol oxidoreductase and iridoid cyclase were overexpressed from a vector, we observed 6 mg/L of nepetalactol in the host (Yee et al., 2019). The S25 strain represents a starting point for our engineering efforts to produce complex MIA *de novo*. Our general strategy was to first increase nepetalactol titer, using three routes: (1) integration of MLPL involved in cyclization; (2) use CRISPR interference (CRISPRi) approach to identify yeast mitochondrial genes that can be inactivated to achieve higher titer; and (3) overexpression of yeast genes involved in mitochondria biogenesis. Following optimization of nepetalactol titer, the remaining pathway and accessory genes shown in Fig. 1 were integrated into the host to assess strictosidine titer. This was followed by expression of genes specific for either heteroyohimbine and corynantheine alkaloid biosynthesis.

**Table 1.** Yeast strains used in this study

Strain	Parent	Genome modifications to parent	Reference
BY4742	S288C	*MATα his3Δ1 leu2Δ0 ura3Δ0 lys2Δ0*	ref (Brachmann et al., 1998) (Brachmann et al., 1998)
DHY214	BY4742	*SAL1^+^ CAT5(91 M) MIP1(661T) MKT1(30 G) RME1(INS-308A) TAO3(1493Q) HAP1+*	ref (Harvey et al., 2018)
JHY651	DHY214	*MATα prb1Δ pep4Δ*	*ibid.*
X303-1B	W303	*MATα ADE2 TRP1 ura3∆0 leu2-3,-112 his3-11,-15* *CAN1 MIP1(661T) SSD1+*	*ibid.*
CEN.PK2-1C	n/a	*MATa; his3D1; leu2-3_112; ura3-52; trp1-289; MAL2-8c; SUC2*	ref (Entian & Kötter, 2007)
S25	JHY651	*ura3Δ::GAL10p-nCox4-ERG13-ADH1t;GAL1p-nCox4-ERG10-CYC1t* *X-2Δ::GAL10p-nCox4-ERG12-ADH1t;GAL1p-nCox4-tHMG1-CYC1t* *HOΔ::GAL10p-nCox4-ERG19-ADH1t;GAL1p-nCox4-ERG8-CYC1t* *rox1Δ::GAL10p-nCox4-IDI1-ADH1t;GAL1p-nCox4-mFPS-CYC1t* *YPRCTy1–2Δ::GAL1p-nCox4-ObGES-CYC1t* *oye2Δ::GAL1p-CrG8H-CYC1t* *oye3Δ*	ref (Yee et al., 2019)
S35	S25	*oye3Δ::ADH2p-CrGOR-PRM9t;PCK1p-CrISY-CPS1t; MLS1p-NmMLPL-SPG5t* *pdr5Δ::mCherry* *cis1Δ::GAL2p-NmMLPL-ADH1t*	*This study*
S36	S35	*pic2Δ::hygR*	*This study*
S37	S35	*ypr011cΔ::hygR*	*This study*
S38	S37	*ypr011cΔ::GAL1p-HAP4-CYC1t*	*This study*
S39	S38	*pic2Δ::hygR*	*This study*
S40	S39	*pic2Δ::GAPp-NmMLPL-ADH1t*	*This study*
yMD015	S40	*iai11 Δ::TEF1p-CPR-PRM9t; PGK1p-CYB5-SPG5t; TDH3p-CYPADH-CYC1t* *egh1Δ::ADH2p-IO-SPG5t;ICL1p-7DLH-PRM9t; PCK1p-SLS-CPS1t* *atf1 Δ::ICL1p-7DLGT-IDP1t; PCK1p-LAMT-CPS1t; bayADH2p-STR-ADH1t*	*This study*
yMD017	yMD015	*xi-5 Δ::ADH2p-TDC-PRM9t;bayADH2p-ZWF1-SPG5t;ICL1p-SAM2-CPS1t*	*This study*
yMD032	yMD017	*ydr514c::PCK1p-RsSGD-SPG5t; ICL1p-CrHYS-CPS1t;bayADH2p-CrSS-CYC1t*	*This study*
yMD034	yMD015	*ydr514c::PCK1p-RsSGD-SPG5t; ICL1p-CrHYS-CPS1t;bayADH2p-CrSS-CYC1t*	*This study*
yJM025	JHY651	*oye3Δ::TEF1p-CPR-PRM9t; PGK1p-CYB5-SPG5t;TDH3p-CYPADH-CYC1t* *yprcty1-2Δ::ICL1p-7DLGT-IDP1t, PCK1p-LAMT-CPS1t, bayADH2p-STR-ADH1t*	ref (Misa et al., 2022)

### Optimization of a De Novo Nepetalactol Producing Strain

**Fig. 2:**
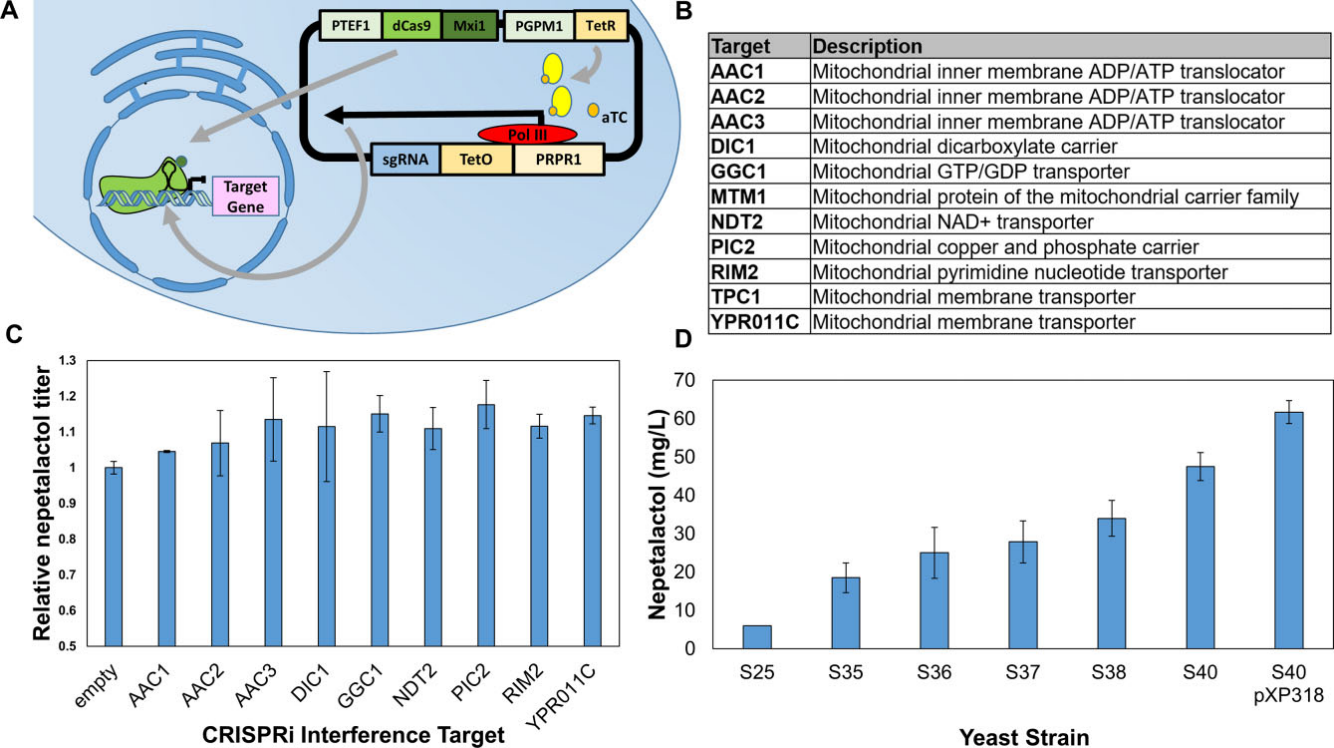
Optimization of nepetalactol titers in *S. cerevisiae.* (**A**) CRISPR-dCas9 interference (CRISPRi) scheme. The guide RNA (sgRNA) targeting the yeast gene is expressed upon addition of anhydrotetracycline (aTC); (**B**) Table of CRISPRi targets tested in yeast strain S35; (**C**) Relative nepetalactol titer from the plasmid-based CRISPRi screen in strain S35 including ‘empty’ control in which S35 contains the CRISPRi plasmid construct with no sgRNA. Titers of biological triplicates of each sample were measured 96 hr post induction; (**D**) Nepetalactol production of modified strains quantified using GC-MS in biological triplicates after 96 hr of growth in galactose-containing media. The PIC2 (S36) and YPR011C (S37) deletion strains were compared to the parent strain S35, final S40 (integrated constitutive overexpression of MLPL and HAP4 yeast gene), and S40 with relieved uracil auxotroph with the plasmid pXP318.

We first integrated GOR and ISY from *C. roseus* and the major latex protein (MLPL) from *Nepeta mussinii* under late stage inducible ADH2-like promoters in the OYE3 locus of S25. We chose to integrate into this locus as previous work from our lab demonstrated knocking out this yeast gene increases the 8-oxogeranial pool by preventing unproductive ‘ene’ reactions (Billingsley et al., 2017). Major latex protein was shown by the O'Connor group to facilitate nepetalactol synthesis through stereoselective cyclization after reduction of 8-oxogeranial by ISY (Lichman et al., 2020). The resulting strain S35 (Table 1) produced 18 mg/L nepetalactol, a threefold increase from our previous published titer without MLPL (Fig. 2D) (Yee et al., 2019).

The S25 strain was designed to synthesize GPP only in the mitochondria. However, we previously observed the unexpected increase in cytosolic geraniol production upon episomal expression of cytosolic geraniol synthase (GES) (Yee et al., 2019). This suggested that a fraction of the diphosphate precursors (isopentenyl pyrophosphate (IPP), dimethylallyl pyrophosphate (DMAPP), or GPP) derived from the mitochondria mevalonate pathway may be transported into the cytosol. In the absence of cytosolic GES, these precursors can be converted to FPP by FPPS and lower the monoterpene yield (Fischer et al., 2011). We hypothesized repression of the unidentified mitochondria transporters in S35 may decrease export of pathway intermediates from the mitochondria, thus increasing the titer of nepetalactol production. Searching through the yeast genome revealed a number of potential transporters that are proposed to transport phosphate-containing metabolites (Fig. 2B). These include the mitochondrial transporters AAC1, AAC2, AAC3 (ATP/ADP) (Bertholet et al., 2022), DIC1(dicarboxylate/phosphate) (Palmieri et al., 1999), GGC1 (GTP/GDP) (Vozza et al., 2004), NDT2 (NAD) (Feitosa-Araujo et al., 2020), PIC2 (copper and phosphate) (McCann et al., 2022), RIM2 (pyrimidine) (Yoon et al., 2011), and YPR011C (adenosine 5’-phosphosulfate, APS, and 3’-phospho-adenosine 5’-phosphosulfate, PAPS) (Todisco et al., 2014). To rapidly identify potential transporter(s) that may be involved in isopentyl-diphosphate transport, we employed a vector-based CRISPRi system to downregulate gene candidates, followed by nepetalactol titer measurement. This CRISPRi system reported by St. Onge and coworkers uses a plasmid containing gene cassettes for constitutive expression of the dCas9-Mxi1 fusion protein and TetR repressor, as well as inducible expression of the target-specific guide RNA (sgRNA) by anhydrotetracycline (aTC) (Fig. 2A) (Smith et al., 2016). Upon induction of the system with aTC to relieve TetR repression of the sgRNA, CRISPRi-dCas9 mediated gene silencing can take place. For the negative control, S35 was transformed with a plasmid expressing dCas9 and TetR but no sgRNA.

Individual yeast transformants were cultured followed by extraction and detection of nepetalactol on GCMS after 72 hr of growth in rich media. Comparing the CRISPRi variants with the control strain revealed repression of the mitochondrial transporters PIC2 and YPR011C led to 14% and 17% increases in nepetalactol titer compared to the negative control (Fig. 2C). There were no considerable differences in nepetalactol titers for other targets. Subsequent quantitative real-time polymerase chain reaction (qRT-PCR) analysis showed PIC2 and YPR011C were repressed four and fivefold by CRISPRi, respectively (Supplementary Table S4). To further validate these targets, we performed individual deletions of PIC2 and YPR011C in S35 to give strains S36 and S37, respectively. Compared to S35, S36, and S37 displayed 35% and 50% increases in nepetalactol production, respectively (Fig. 2D). These results implicate YPR011C and PIC2 may facilitate export of diphosphate intermediates from the mitochondria to cytosol, although further characterization is required. Recently, Pagliarini and coworkers identified Hem25p (Ydl119cp), a mitochondrial glycine carrier, as the primary IPP transporter into the mitochondria, which may be an additional target for silencing (Tai et al., 2023).

Next, we investigated the effect of mitochondrial transcription factor overexpression on nepetalactol production in S35. We chose to overexpress the transcription factor HAP4 because this activator facilitates the diauxic shift, increasing cellular respiration and mitochondrial biogenesis (Shi et al., 2018). We hypothesized increased production of mitochondria may lead to higher production of monoterpene precursors since the engineered geraniol pathway is localized to the mitochondria. The overexpression cassette for HAP4 was integrated into S35 at the YPR011C locus identified above to generate S39 (Table 1), which led to a 22% increase in nepetalactol titer compared to the strain with deletion in YPR011C (S38) (Fig. 2D). Following this, PIC2 was deleted in S39 (Table 1), but unexpectedly, no synergistic increase in nepetalactol titer was observed (Fig. 2D). Nevertheless, we proceeded with S39 and integrated an additional copy of MLPL under the constitutive GAP promoter in the PIC2 locus to arrive at S40 (Table 1), which led to a 40% increase in nepetalactol production (Fig. 2D). Higher levels of constitutive MLPL expression therefore further decrease shunt product formation during conversion of 8-oxogeranial to nepetalactol, resulting in increased titer.

Throughout this work, we observed strains transformed with vectors with the URA3 marker produced higher levels of nepetalactol. This led to the hypothesis that relieving the URA3 auxotrophy may improve overall cell health and lead to greater strain productivity. To test the effect of URA3 expression on titer, a direct comparison of nepetalactol production in S40 with and without an empty low copy URA3 plasmid (pXP318) was performed. S40 transformed pXP318 indeed showed a 30% increase in nepetalactol titer (62 mg/L) (Fig. 2D). The nepetalactol titer of S40/pXP318 is 10.5-fold higher compared that reported in our previous work. We did not relieve the auxotroph of this strain at the genome level as URA3 is highly useful for episomal expression of pathway genes.

### Generation of a De Novo Strictosidine Producing Strain

**Fig. 3:**
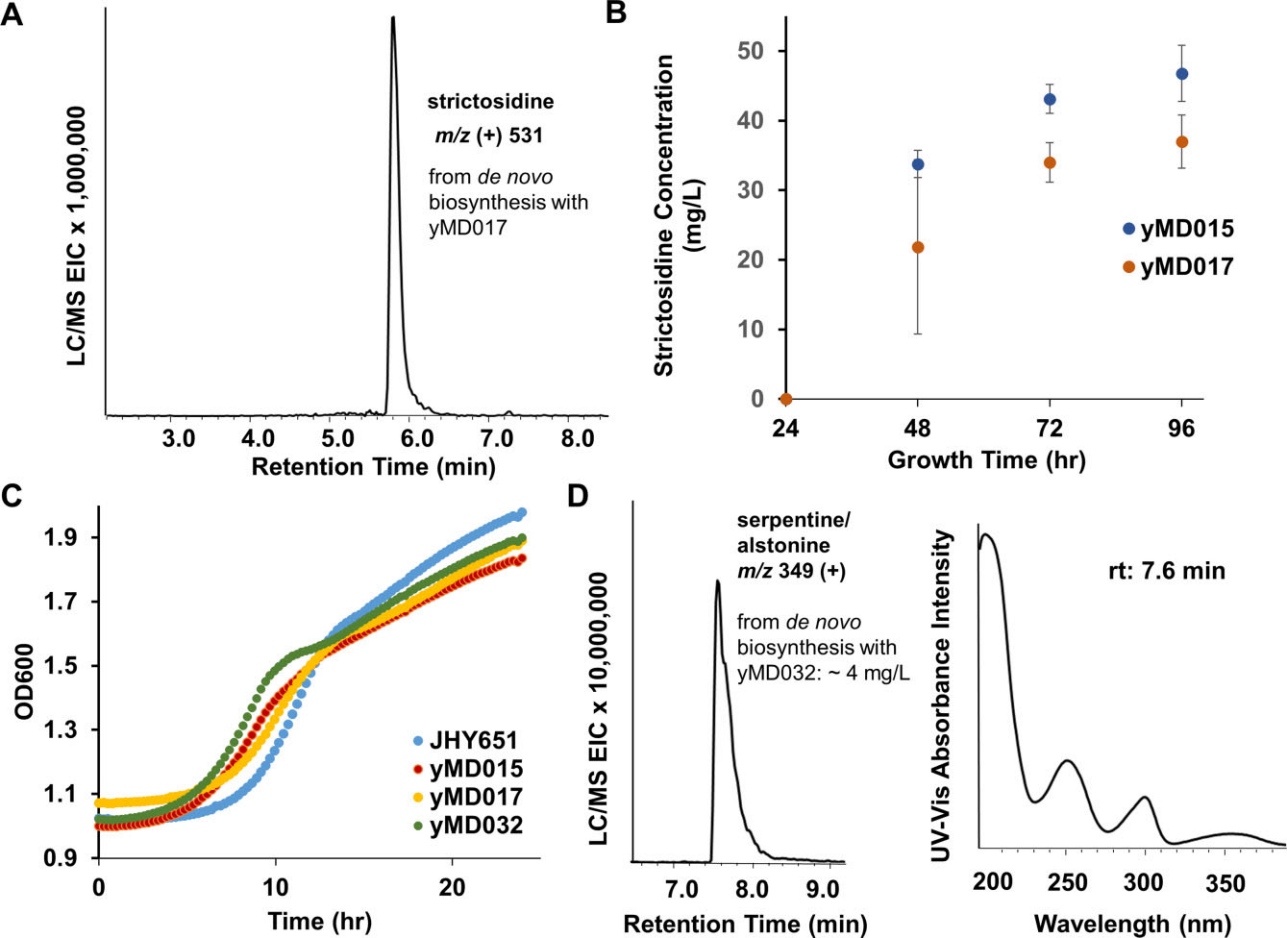
Biosynthesis of strictosidine and serpentine from *S. cerevisiae.* (**A**) LCMS trace (selected ion monitoring at *m/z* (+) = 531) of strictosidine produced from yMD017 after 72 hr growth in 1.9 % gal 0.5 % glu media. (**B**) Time course analysis of strictosidine production from yMD017 and yMD015. The latter strain does not express tryptamine decarboxylase and was supplemented with 2 mM tryptamine after 24 hr; (**C**) Growth curves of yeast strains compared against the starting strain JHY651. (**D**) LCMS trace (selected ion monitoring at *m/z* (+) = 349) of serpentine and alstonine production from yMD032 after 96 hr of growth. The titer was estimated to be 4.9 mg/L based on comparison to standards. The UV-vis absorbance is consistent with that report for serpentine, as well as that measured for an authentic standard (rt 7.6 min).

To generate a strain for *de novo* strictosidine production, we chose to integrate into the IAI11, EGH1, and ATF1 loci, as these have precedence as yeast genomic knockout sites for terpene pathway integration (Brown et al., 2015; Srinivasan & Smolke, 2020). IAI11 encodes a putative mitochondrial protein with unknown function that we have observed is nonessential for robust yeast metabolic function. EGH1 encodes a glucosidase, which we reasoned could prevent nonspecific deglucosylation of the glucosyl protection group on intermediates following 7DLGT function in the pathway (Fig. 1). ATF1 encodes an alcohol acetyltransferase that can acetylate geraniol, and knockout of this yeast gene has previously been shown to improve strictosidine titers (Brown et al., 2015). The cytochrome P450 accessory enzyme genes CPR, CYB5, and CYPADH from *C. roseus* were cloned under strong constitutive promoters and integrated in the IAI11 locus in strain S40. Next, genes encoding the cytochrome P450 monooxygenases IO, 7DLH, and SLS from *C. roseus* were integrated into the EGH1 locus under the autoinducible ADH2-like promoters (Harvey et al., 2018). Finally, genes encoding 7DLGT, loganic acid O-methyltransferase (LAMT), and STR placed under ADH2-like promoters were integrated into the ATF1 locus to give a plasmid-free strain for strictosidine production, yMD015. This strain was cultured in galactose and glucose containing media and supplemented with 2 mM tryptamine following 48 hr of growth. Production of strictosidine was detected by liquid chromatography mass spectroscopy (LCMS) after 72 additional hours of growth. The identity of strictosidine was matched to purified standard from previous work (Misa et al., 2022) and the concentration was rigorously measured using TripleQuad mass spectrometry following calibration with standards (Supplementary Fig. S1A). The galactose and glucose concentrations of the rich media were then varied to measure the effect on strictosidine titers (Supplementary Fig. S1B). As shown in Fig. 3B and Supplementary Fig. S1, when the rich media contains 1.9% galactose and 0.5% glucose, the titer of strictosidine was the highest at 43 mg/L 96 hr after tryptamine addition. This yMD015 strain therefore produced strictosidine *de novo* at titers similar to the geraniol-fed strain previously described (Misa et al., 2022), and can be used to produce strictosidine and MIA analogs using substituted tryptamine analogs.

To arrive at a complete *de novo* strain without tryptamine feeding, the gene encoding tryptophan decarboxylase from *C. Roseus* (CrTDC), together with overexpression cassettes of two yeast genes ZWF1 and SAM2, were integrated into the XI-5 locus (Mikkelsen et al., 2012) under ADH2-like promoters in yMD015 to arrive at strain yMD017 (Table 1). We chose to integrate into the XI-5 locus, an intergenic region in the yeast chromosome, to decrease the chances of disrupting the function of nearby essential genes. This site was reported by Mikkelson and co-workers to be an optimal site for integration due to its demonstrated support of high heterologous gene expression with minimal impact on cellular fitness (Mikkelsen et al., 2012). SAM2 is involved in biosynthesis of *S*-adenosyl-L-methionine (SAM), which is required for the methyltransferase activity of LAMT, and ZWF1 expression increases NADPH availability which is required in the redox reactions in the pathway (Fig. 1) (Chu et al., 2013; Kwon et al., 2006) Integration of both genes have been shown by O'Connor and coworkers to improve strictosidine in the first demonstration of *de novo* production (Brown et al., 2015). The strain yMD017 reached the highest titer of strictosidine at ∼34 mg/L in the galactose-rich media after 96 hr of growth without feeding additional substrates (Figs 3A and B).

The fitness of yMD015 and yMD017 were measured by monitoring the optical density of the cultures in galactose-rich media for 24 hr post-inoculation. While small differences in final OD were observed in these strains compared to the parent JHY651, the abundance of genetic modifications did not result in significant growth retardation (Fig. 3C). Prolonged culturing of these strains also did not lead to significant differences in final cell density. Therefore, we successfully engineered a fully *de novo* yeast strain that produces the universal MIA intermediate strictosidine without compromising cellular fitness. The titer of strictosidine is similar to that which relies on geraniol feeding. However, unlike the biotransformation strain that requires 2 mM of geraniol precursor, no biosynthetic intermediates or shunt products can be detected from the *de novo* strains, underscoring the advantage of drawing precursor flux from primary metabolism compared to exogenous feeding.

### De Novo Biosynthesis of Heteroyohimbine Alkaloids in Yeast

To demonstrate the applicability of our *de novo* strictosidine-producing strains, yMD015 and yMD017, for production of bioactive MIAs, we targeted the biosynthesis of serpentine and alstonine, which belong to the heteroyohimbine family of MIAs (Stavrinides et al., 2016). Hydrolysis of the glucose protection group in strictosidine by SGD reveals the strictosidine aglycone. The HYS discovered from *C. roseus* reduces the cathenamine form of strictosidine aglycone to a mixture of 20*S* ajmalicine and 20*R* tetrahydroalstonine (Stavrinides et al., 2016), while the P450 SS can oxidize these into the indoloquinolizidine serpentine and alstonine, respectively (Supplementary Fig. S5) (Yamamoto et al., 2021). The cassette of genes encoding SGD from *Rauvolfia serpentina* (Zhang et al., 2022), HYS and SS, under the control of ADH2-like promoters, were integrated into the YDR514C locus of yMD015 and yMD017 to give yMD034 and yMD032, respectively (Table 1). Pyne and coworkers chose this site as one of the seven oxidoreductase encoding sites to disrupt in their tetrahydroisoquinoline alkaloid production platform, and found the inactivation of this gene to improve titers (Pyne et al., 2020). We have previously seen that deletion of ARI1 and ADH6, functionally analogous to YDR541C, improve iridoid titers by preventing unproductive oxidoreduction reactions on aldehyde containing intermediates (Billingsley et al., 2017). The growth curve of yMD032 in rich yeast media showed nearly identical robustness to yMD015 or yMD017 (Fig. 3C).

The strain yMD032 was grown in galactose-rich media and extracted after 96 hr without any substrate feeding. LCMS analysis of extracts showed the emergence of a new peak (*m/z* (+) = 349) (Fig. 3D) with retention time (rt) and MS/MS fragmentation pattern (Supplementary Fig. S5) matching both (1) serpentine generated from biotransformation of ajmalicine by yeast strain (yJM025) expressing only SS (using plasmid pMD019). (Supplementary Fig. S2); and (2) serpentine hydrogen tartrate purchased from commercial vendor (Supplementary Fig. S4). The UV-vis absorbance of the compound produced by yMD032 also matches that reported in literature (Fig. 3D) (Stavrinides et al., 2016). Furthermore, upon feeding of 7-fluorotryptamine, we detected comparable titer of a new compound with *m/z* (+) = 367, which matches to that expected for fluoro-serpentine (Supplementary Fig. S3). Based on this data, we concluded that a combination of serpentine and alstonine is biosynthesized by yMD032. Using a calibration curve generated from commercial serpentine tartrate, the titer of serpentine/alstonine was estimated to be ∼4.9 mg/L after 96 hr of growth (Supplementary Figs S4 and S6). Since no reductase specific for ajmalicine is known, it is currently not possible to afford exclusively serpentine starting from strictosidine. The enzyme, tetrahydroalstonine synthase (THAS), is a stereospecific 20*R* reductase that gives tetrahydroalstonine but not ajmalicine (Stavrinides et al., 2016). However, when HYS in yMD032 was replaced with THAS, the signal of the *m/z* (+) = 349 peak was significantly lower and no UV signal that matches alstonine can be detected due to the low titer. From yMD032, we observed strictosidine production on day 2 of culturing. By day 4 when serpentine/alstonine was at its peak levels, accumulation of strictosidine was no longer detected, indicating the intermediate is consumed by the downstream enzymes. Lastly, we attempted expression of the sarpagan bridge enzyme from *R. serpentina* (Dang et al., 2018) in place of HYS, but the resulting strain did not produce any new compounds.

### De Novo Biosynthesis of Corynantheine Alkaloids in Yeast

We further tested the use of yMD015 or yMD017 as a starting point for reconstituting the corynantheine-family of alkaloids. Recent work by O'Connor and coworkers characterized the initial steps of mitragynine pathway starting from the corynantheine iminium form of the strictosidine aglycone (Fig. 1), which includes reduction catalyzed by MsDCS1 and methylation by MsEnoMT4 (Schotte et al., 2023). The remaining hydroxylation and methylation steps have not been elucidated to date. The O'Connor and Qu labs have used different strategies to complete the mitragynine pathway despite the missing enzymes, albeit both at trace titers (Kim et al., 2023; Schotte et al., 2023).

**Fig. 4:**
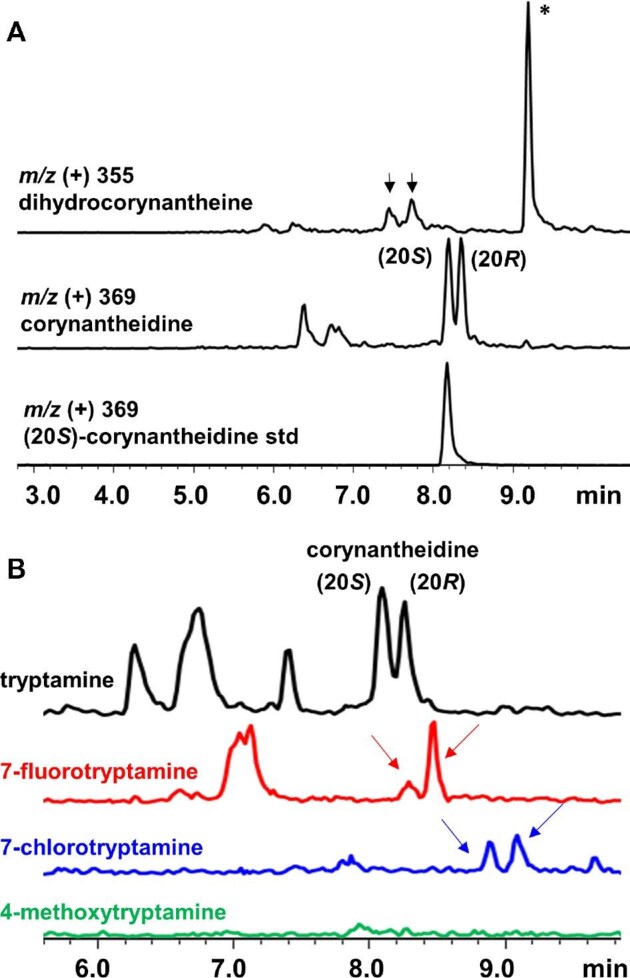
Biosynthesis of corynantheine alkaloids in the mitragynine pathway from *S. cerevisiae.* (**A**) LCMS trace (selected ion monitoring of *m/z* as shown) of dihydrocorynantheine and corynantheidine produced from strain yMD032 expressing MsDCS1 and MsEnoMT4. The standard of (20S)-corynantheidine is shown in the bottom trace; (**B**) LCMS trace (selected ion monitoring) of corynantheidine and analogs detected from *S. cerevisiae* as a result of feeding tryptamine or tryptamine analogs. The arrows indicate peaks associated with targeted compounds, and the asterisk indicates endogenous yeast peak unrelated to the biosynthetic pathway.

The strain yMD015 was transformed with the plasmid pJM126, which contains RsSGD, MsDCS1, and MsEnolMT4 expressed under ADH2 promoters. Biological triplicates of these transformants were fed tryptamine to a final concentration of 2 mM after 24 hr of outgrowth and later extracted 96 hr following feeding. LCMS analysis of extracts showed the emergence of four new peaks, two with *m/z* (+) = 355 and two with *m/z* (+) = 369, putatively corresponding to both isomers of dihydrocorynantheine and corynantheidine, respectively (Fig. 4A). The later rt of the *m/z* (+) = 369 pair is consistent with a methyl addition. Removal of MsEnolMT4 resulted in the abolishment of the corynantheidine diastereomeric pair and only the putative dihydrocorynantheidine pair remained. Comparison to an analytical standard of (20*S*)-corynantheidine confirmed the same rt to one of the peaks with *m/z* (+) = 369. Using a standard curve generated with purified (20*S*)-corynantheidine, we estimated the (20*S*)-corynantheidine titer to be 0.9 ± 0.1 mg/L after 96 hr of growth (Supplementary Fig. S7).

The significant drops in titer from strictosidine to both the serpentine and corynantheidine are most likely attributed to the low efficiency of RsSGD in *S. cerevisiae*, as observed by Keasling and coworkers in their reconstitution of the iboga MIAs (Zhang et al., 2022). Strictosidine β-D-glucosidase contains a nuclear localization tag and is shown to be localized into the nucleus in plant cells (Stavrinides et al., 2015, 2016). The role of this unusual localization is not clear but has been hypothesized to sequester the reactive strictosidine aglycon after removal of the reactive glucose moiety (Barleben et al., 2007). The ring-opened form of the aglycone contains both an electrophilic aldehyde and a nucleophilic enol which can result in significant cellular toxicity (Stavrinides et al., 2015). Downstream enzymes such as HYS and MsDCS1 have also been shown to be colocalized with SGD in the nucleus to further tame the reactive aglycon (Stavrinides et al., 2016; Wu et al., 2023). The inefficiency of the post-strictosidine steps observed in the reconstitution work may therefore be due to differences in cellular physiology between yeast and plant cells and represents a bottleneck for high-titer production of complex MIAs in yeast.

Lastly, to test if analogs of corynantheidine can be accessed through feeding of tryptamine analogs using the yMD015/pJM126 strain, we supplied the growth culture with 7-fluorotryptamine, 7-chlorotryptamine and 4-methoxytryptamine. The cultures were extracted 72 hr after feeding and analyzed by LCMS using selected ion monitoring (Fig. 4B). Both the fluorinated and chlorinated adducts of corynantheidine pair can be detected with the expected shifts in retention time. In the case of 7-chlorotryptamine feeding, significantly lower MS intensities of the selected ions were observed. No accumulation of the 4-methoxy analogs, mitragynine, and speciogynine, were detected under our assay conditions. In the absence of the last two enzymes in mitragynine pathway, alternative strategies such as incorporating tryptamine 4-hydroxylase and the newly discovered 4-hydroxyindole methyltransferase, as reported by Qu and coworkers, could be adopted to access mitragynine from our yeast platform (Kim et al., 2023).

## Methods

### Plasmid and Strain Construction

Yeast expression vectors were constructed using yeast homologous recombination. CRISPRi plasmids were assembled through Gibson Assembly. CRISPRi guide RNA sequences were designed using Yeast CRISPRi (http://lp2.github.io/yeast-crispri/). Q5® High-Fidelity DNA Polymerase New England Biolabs (NEB) and Phusion (NEB) were used for PCR. The cassette for expression of remaining nepetalactol genes, GOR, ISY, and MLPL, the p450s, IO,7DLH, and SLS, the remaining strictosidine genes, 7DLGT, LAMT, and STR, tryptophan decarboxylase and the cofactor regeneration genes, TDC, ZWF1, SAM2, and the post-strictosidine genes towards serpentine, SGD, HYS, SS, all under ADH2-like promoters, were amplified from pJB204, pJM057, pJM029, pMD034, and pMD050, respectively. The ADH2-like promoters, constitutive promoters, and the HAP4, ZWF1, and SAM2 genes were amplified from *S. cerevisiae* genomic DNA. The ADH2 homologue “bayADH2p” is from *Saccharomyces bayanus.* The reductase partners, CPR, CYB5, and CYPADH, under constitutive promoters were amplified off pJB153. The primers and plant genes sequences used in this study were ordered from Integrated DNA Technologies. Purchased gBlocks were codon-optimized for expression in *S. cerevisiae.* Primers used in this study are listed in Supplementary Table S1. Plasmids were verified by Sanger sequencing and were maintained and propagated in *Escherichia coli* TOP10. Plasmids used in this study are listed in Supplementary Table S2. All yeast transformations were performed using the lithium acetate PEG method (Gietz & Schiestl, 2007). Deletions of yeast genes were achieved by either integration of the hygromycin resistance gene (hygR) containing an upstream *Sce*I restriction site with 50–100 bp homology to the integration site or a LEU2 selective marker with 50–100 bp homology to the integration site. Genes were integrated into the hygromycin or LEU2 landing pad though co-transformation of a plasmid with the G418 marker expressing *Sce*I for hygR-based knockout or expressing CRISPR-Cas9 machinery and sgRNA targeting LEU2 gene as well as donor DNA containing the integration cassette with 50–100 bp homology to the integration site (Horwitz et al., 2015). Yeast transformants were recovered in standard yeast extract peptone dextrose (YPD) media for 16 hr and then plated on YPD agar plates with 200 mg/L hygromycin or synthetic defined (SD) 2% glucose agar plates with amino acids minus LEU2 for knockouts or with G418 sulfate for GOI integrations. Yeast colonies were screened by colony PCR followed by Sanger sequencing of PCR amplicons of the integration loci.

### Culture Conditions

For fully integrated strains, strains were streaked onto YP agar plates with 4% glycerol and single colonies of each construct were inoculated in 1 mL YPD. For plasmid bearing strains, including the CRISPRi transformants and pXP318 containing strain, single colony transformants were inoculated in 1 mL SD 2% glucose media with the appropriate dropouts. Starter cultures were shaken at 28°C and 250 rpm for 16–24 hr. For CRISPRi yeast transformants and strains yDY035-yDY040, culture tubes containing 3 mL of YP 0.05% glucose 3.8% galactose were inoculated with 100 μL of starter culture. For strains yMD015-yMD032, culture tubes containing 3 mL of YP 0.5% glucose 1.9% galactose were inoculated with 100 uL of starter culture. For CRISPRi assays, cultures were supplemented with 200 mg/L G418 sulfate and 250 μg/L anhydrotetracycline at the beginning of the assay, 24 hr after outgrowth in production media. For yMD015, cultures were supplemented with 2 mM tryptamine 24 hr after outgrowth in production media. Subcultures were shaken at 28°C and 250 rpm for 24–120 hr before extraction for metabolite analysis.

### Growth Assays

All strains were grown overnight in biological triplicate in 1 mL YPD or respective selective media. These overnight cultures were used to inoculate 100 μL of YPD to a starting OD_600_ of 0.01 in a 96-well clear plate. The plate was then sealed and placed into an Infinite M200 plate reader (TECAN) for incubation. Cultures were continuously shaken at 280 RPM at 28°C with OD_600_ measurements taken every 15 min for 24 hr.

### Culture Extraction and Quantification

For quantification of geraniol production, the two-phase subcultures were centrifuged at 4300 rpm at 20°C for 6 min to separate the dodecane and aqueous layers. Nepetalactol and corynantheidine production were measured by extracting 200 μL subculture with 200 μL of an organic phase consisting of 25% acetone and 75% ethyl acetate. The samples were vortexed for 1 min then centrifuged for 10 min. The organic layers were analyzed on an Agilent Technologies GC-MS 6890/5973 equipped with a DB-FFAP column. An inlet temperature of 220°C and constant pressure of 4.2 psi were used. The oven temperature was held at 60°C for 5 min and then ramped at 60°C/min for 1.5 min, followed by a ramp of 15°C/min for 16 min and a hold for 10 min. All measurements were taken in biological triplicate unless otherwise noted. Standard curves were generated using purified nepetalactol and corynantheidine (Supplementary Fig. S7).

For quantification of strictosidine and serpentine production, samples were extracted 24–120 hr after feeding substrates. A volume of 200 μL of whole culture was extracted with 200 μL methanol and vortexed for 30 s. The samples were then centrifuged for 10 min at maximum speed. The supernatant is then separated from solid pellet for proceeding analysis. Strictosidine samples were analyzed on Thermo Fisher TSQ Altis inline triple quadrupole mass spectrometer coupled with an ultra-high-performance liquid chromatograph (UHP LCMS/MS) equipped with a Phenomenex Kinetex C18, 1.7 μm, 100 Å, 2.1 × 100 mm reverse-phase column. Positive mode electrospray ionization was performed with a linear gradient of 5–65% acetonitrile-H_2_O spiked with 0.1% formic acid over 15 min and then 95% acetonitrile for 3 min with a flow rate of 0.3 mL/min. Serpentine samples were analyzed on a Shimadzu 2020 EV LCMS equipped with a Phenomenex Kinetex C18, 1.7 μm, 100 Å, 2.1 × 100 mm reverse-phase column. Both positive- and negative-mode electrospray ionization were performed with a linear gradient of 5–95% acetonitrile-H_2_O spiked with 0.1% formic acid over 15 min and then 95% acetonitrile for 3 min with a flow rate of 0.3 mL/min. Standard curves were generated using purified strictosidine from previous work (Misa et al., 2022) and commercial serpentine hydrogen tartrate, respectively (Supplementary Figs S1, S4).

### qRT-PCR Analysis

For qRT-PCR analysis of CRISPRi repression of PIC2 and YPR011C, 500 μL of culture was harvested after 48 hr of growth in the culturing conditions described above. The samples were centrifuged and the supernatant was discarded. RNA was extracted from the cell pellets using RiboPure™ Yeast RNA Isolation Kit (Ambion) following the manufacturer's instructions. Residual genomic DNA in the extracts was digested by DNase I (2 U/μL) (Invitrogen) at 37°C for 8 hr. SuperScript III First-Strand Synthesis System (Invitrogen) was used for cDNA synthesis with oligo-dT primers following directions from the user manual. The synthesized cDNA was diluted 1:50 and then used for SYBR qRT-PCR. Quantitative real-time PCR primers were designed using PrimerQuest^TM^ from IDT to give products of 100 base pairs. Primer sequences are listed in Supplementary Table S. Quantitative real-time PCR was performed using iQ™ SYBR^®^ Green Supermix and the CFX96 Touch Real-Time PCR Detection System.

## Conclusions

We engineered a fully integrated yeast strain that reliably produces ∼30–40 mg/L strictosidine *de novo* after 96 hr of growth. Our strain is based on the mitochondria compartmentalization strain previously constructed to achieve high titers of monoterpene precursor. The yMD015 and yMD017 pair is suitable for reconstitution of complex MIA pathways, as exemplified by the biosynthesis of heteroyohimbine and corynantheine alkaloids, and fluorinated analogs thereof. Further engineering efforts are underway to overcome the noted bottlenecks in steps immediately following strictosidine biosynthesis.

## Supplementary Material

kuad047_Supplemental_File
