# Supplementary material for: Engineered biosynthesis of plant heteroyohimbine and corynantheine alkaloids in Saccharomyces cerevisiae
Source: J Ind Microbiol Biotechnol. 2023 Dec 22;51:kuad047. doi: 10.1093/jimb/kuad047 (PMC10995622; doi:10.1093/jimb/kuad047)
Supplement: kuad047_Supplemental_File [file kuad047_supplemental_file.pdf]

# Supporting Information

## Engineered Biosynthesis of Plant Heteroyohimbine and Corynantheine Alkaloids in *Saccharomyces cerevisiae*

Moriel J. Dror[1,2], Joshua Misa[1], Danielle A. Yee[1], Angela M. Chu[3], Rachel K. Yu[1,4], Bradley B. Chan[1,2], Lauren S. Aoyama[1], Anjali P. Chaparala[1], Sarah E. O'Connor[5], Yi Tang[1,6,*]

*[1]Department of Chemical and Biomolecular Engineering, [2]Department of Bioengineering, [4]Department of Molecular Cell and Developmental Biology, [6]Department of Chemistry and Biochemistry, University of California, Los Angeles, Los Angeles, CA,90095, USA*
*[3]Stanford Genome Technology Center, Stanford University, Stanford, CA,94305, USA*
*[5]Department of Natural Product Biosynthesis, Max Planck Institute for Chemical Ecology, Jena, 07745, Germany*

Correspondence to:
Prof. Yi Tang
Department of Chemical and Biomolecular Engineering
University of California, Los Angeles
Los Angeles, 90095, USA
TEL: +1-(310) 825-0375 E-MAIL: yitang@g.ucla.edu

# Table of Contents

**Supplementary Tables**

**Table S1:** Primer sequences used in this study **S3-S4**
**Table S2**: Plasmids used in this study **S5**
**Table S3**: CRISPRi gRNA targets **S6**
**Table S4**: qRT-PCR calculations **S6**

**Supplementary Figures**

**Figure S1**: Strictosidine standard curve and media optimization **S7**
**Figure S2**: Serpentine biosynthesis from ajmalicine **S8**
**Figure S3**: *De novo* synthesis of 7-fluoro-serpentine **S9**
**Figure S4**: Serpentine hydrogen tartrate standard MS trace **S10**
**Figure S5**: *De novo* serpentine MS/MS spectra compared with standard **S11**
**Figure S6**: Time course of *de novo* serpentine production from yMD032 **S12**
**Figure S7**: Corynanthideine standard curve **S13**

## Supplementary Tables

**Table S1.** Primers (sequence 5’ to 3’)

| Primer Name | Primer Sequence |
|---|---|
| OYE3 ADH2P F | TACAACAACTGTAGTTCAGTATAGCGAAGTTTAAATTTAGAAGAACGCCGCAAAACGTAG |
| SPG5t OYE3 R | AAAAATACATAACATCAATGTCTTTATTCATGATTGCTTATTTTCTGCCGAATTTTCATG |
| OYE3 hygR F | ACAACTGTAGTTCAGTATAGCGAAGTTTAAATTTAGAAGATTACCCTGTTATCCCTAGAC |
| hygR OYE3 R | TCAATGTCTTTATTCATGATTCCACATTAACCTTCTTTGATGGTCAGTATAGCGACCAGC |
| OYE3 F2 | AAAAGTACGTACTTGATATATACAACAACTGTAGTTCAGTATAGCGAAGTTTAAATTTAG |
| OYE3 R2 | TATGTGAGTTTTGTAATTAAAAATATGGCAGGAATATGAAAAATACATAACATCAATGTC |
| PIC2 hygR F | AAGGGAAAAAGAGAACGGACTCATTGACAGTTGTAAAGCATTACCCTGTTATCCCTAGAC |
| hygR PIC2 R | GTGTTTACGATAAACCTTGCCGATTGAATCGATCAGCCACATTAACCTTCTTTGATGGTC |
| YPR011C hygR F | GTATTGACTTTCAAATTTTTTAATCGTTATTTCGCTAACATTACCCTGTTATCCCTAGAC |
| hygR YPR011C R | CTTGGTTTTCGCTGACCCAAGCCTGTCACCTCAAGCCCACATTAACCTTCTTTGATGGTC |
| YPR011C GALp F | TTCAAATTTTTTAATCGTTATTTCGCTAACTTTCAAAAATTCTTACTTTTTTTTTGGATG |
| CYC1t YPR011C R | CATTACTTGGTTTTCGCTGACCCAAGCCTGTCACCTCAAGCCTTCGAGCGTCCCAAAACC |
| pic2 GAPp F | AAAAAGAGAACGGACTCATTGACAGTTGTAAAGCTCATTATCAATACTGCCATTTCAAAG |
| adh1t pic2 R | AGTGTTTACGATAAACCTTGCCGATTGAATCGATCAGCCGGTAGAGGTGTGGTCAATAAG |
| galp hap4 F | TATACCTCTATACTTTAACGTCAAGGAGAAAAAACATGACCGCAAAGACTTTTCTACTAC |
| hap4 cyc1 R | ACTCCTTCCTTTTCGGTTAGAGCGGATTCAAAATACTTGTACCTTTAAAAAATCGACATC |
| pJB097 GAPp F | TAGGCGTATCACGAGGCCCTTTCGTCTGTCATTATCAATACTGCCATTTCAAAGAATACG |
| XhoI-SceI REV | CTCGAGTTATTTCAGGAAAGTTTCG |
| YPR011C RT FWD Set 1 | GTTGTAGTGTGGTGGCTACTT |
| YPR011C RT REV Set 1 | CCCGGAGGCTTTGATATACTTT |
| PIC2 FWD Set 1 | GGTGCTACATTCGTCGGATATT |
| PIC2 REV Set 1 | CACAGTGACACCAGGACTTAAC |

| | |
|---|---|
| ACT2 qRT-PCR FWD Set 1 | TCGAAACTGTGCGTCAGATAAA |
| ACT2 qRT-PCR REV Set 1 | CCTGCCATCTGGTAACTCATAC |
| IAI11 Up F | CATTTATCGAGTGCATTGATGAAGTCC |
| IAI11 Up R | ATTTTCTTCATGGCAATTCTACATGTTATAAGTG |
| IAI11 Down F | GATAACTGCAAAAGGTATGCATAGGC |
| IAI11 Down R | GGTTGGTTCAGGAGAGGTAGAACC |
| EGH1 Up F | CACGCTCCCATAGAAAATGAC |
| EGH1 Up R | TCCCTGCCCGTTTTGGGGTC |
| EGH1 Down F | CGCTGCATTGGATGCTCTTGG |
| EGH1 Down R | CTCGTGAAACCATTCAAGAACCT |
| ATF1 Up F | CAAGAAAATAAAAAACGGCACTTCATCAG |
| ATF1 Up R | GAATTCTTCTAATATTTGCTTCATTACTG |
| ATF1 Down F | CCACTTATTGAGCACTACCATG |
| ATF1 Down R | GGGTTATTTACACGACATAATCATATTGTCG |
| XI-5 Up F | GCGGAGAAGTCGTTGATAGC |
| XI-5 Up R | CTGCTTTAGTGTGTAACGTTCTGC |
| XI-5 Down F | GCCTTCGATTTGACACATCTC |
| XI-5 Down R | GATCATAGATCCGGCACTTAGAG |
| YDR541C Up F | GTTTGTTTCTTCTTATCTTCAGCTGCTGAG |
| YDR541C Up R | ACAATAGCTTATAATCTGTGTAGTCAAACTATATACTAGGC |
| YDR541C Down F | ATCTAGCTAGAAGTTTTGTAGGTATATGTGATTTAAGATATAG |
| YDR541C Down R | CATTATCACGTTGTTTGCCACAAGAATTATTG |
| EGH1 ADH2p F | TTACATAATTATTATATTGACCCCAAAACGGGCAGGGAGC CGCAAAACGTAGGGGCAAAC |
| EGH1 CPS1t R | CCTTCTAATGCAAATCCAAGAGCATCCAATGCAGCGATTT GACACTTGATTTGACACTTC |
| ATF1 ICL1p F | ATGAAGCAAATATTAGAAGAATTCATTTATTGAAAAGTAAA TATCTCGTAACCCGGATGC |
| ATF1 ADH1t R | TTAAAGCTTCCGAAATTACTTCATGGTAGTGCTCAATAAGT GGGCATGCCGGTAGAGGTG |
| XI-5 CPS1t R | CTCAGTTTCAGCTTAGAGATGTGTCAAATCGAAGGCATTT GACACTTGATTTGACACTTC |
| XI-5 ADH2p F | ACGAAAGCTAGTCGCAGAACGTTACACACTAAAGCAGCAA AACGTAGGGGCAAACAAACG |
| YDR541C PCK1p F | TATAGTTTGACTACACAGATTATAAGCTATTGTCAATAGGA AAAAACCGAGCTTCCTTTC |
| YDR541C CYC1t R | CTTAAATCACATATACCTACAAAACTTCTAGCTAGATGCAA ATTAAAGCCTTCGAGCGTC |
| IAI11 ADH2p F | ATTTTTACACTTATAACATGTAGAATTGCCATGAAGAAAAT CCGCGAATCCTTACATCAC |
| IAI11 CYC1t R | GAAGTTATTGCCTATGCATACCTTTTGCAGTTATCGCAAAT TAAAGCCTTCGAGCGTCCC |

71

**Table S2.** Plasmids

| Plasmid | Description | Reference |
|---|---|---|
| pJB204 | 2μ yeast ori; URA3; ColE1 ori; AmpR; ADH2p-CrGOR-PRM9t;PCK1p-CrISY-CPS1t; MLS1p-NmMLPL-SPG5t | *ref 33* |
| pJM057 | CEN/ARS yeast ori; HIS3; ColE1 ori; AmpR; ADH2p-IO-SPG5t;ICL1p-7DLH-PRM9t; PCK1p-SLS-CPS1t | *ref 33* |
| pJM029 | 2μ yeast ori; URA3; ColE1 ori; AmpR; ICL1p-7DLGT-IDP1t; PCK1p-LAMT-CPS1t; bayADH2p-STR-ADH1t | *ref 33* |
| pJB153 | 2μ yeast ori; HIS3; ColE1 ori; AmpR; TEF1p-CPR-PRM9t; PGK1p-CYB5-SPG5t; TDH3p-CYPADH-CYC1t | *ref 33* |
| pXP318 | CEN/ARS yeast ori; URA3; ColE1 ori; AmpR; TEF1p- CYC1t | *this study* |
| pMD034 | 2μ yeast ori; URA3; ColE1 ori; AmpR; ADH2p-CrTDC-PRM9t;bayADH2p-ZWF1-SPG5t;ICL1p-SAM2-CPS1t | *this study* |
| pMD050 | 2μ yeast ori; URA3; ColE1 ori; AmpR; PCK1p-RsSGD-SPG5t; ICL1p-CrHYS-CPS1t;bayADH2p-CrSS-CYC1t | *this study* |
| pMD019 | 2μ yeast ori; URA3; ColE1 ori; AmpR; ADH2p-CrSS-SPG5t | *this study* |
| pJM126 | 2μ yeast ori; HIS3; ColE1 ori; AmpR; PCK1p-RsSGD-SPG5t-ICL1p-MsDCS1-PRM9t-PCK1p-MsEnolMT4-CPS1t | *this study* |

**Table S3.**CRISPRi guide RNA targets

| Gene target | Guide RNA sequence (5'to 3') |
|---|---|
| ADH6 | AAAAGCACCAACAGTTCTCG |
| ADH7 | ATAATTTCTACTCAGAGTTT |
| ARI1 | AAGTTGCATAGAATAAATTC |
| AAC1 | TAAAGCTTATTTCTGTCGGA |
| AAC2 | ATTTATATTGTGCGACGACG |
| AAC3 | AAATCTTATATGTAATTTGA |
| DIC1 | GTATGCATATTTATGTGTAG |
| GGC1 | AAATCGTGTATCTCTTTTGA |
| MTM1 | CTATTCTGAGATCTTCAAGA |
| NDT2 | GATTCTCGACATACTCGCGC |
| PIC2 | AAGCAAAATGAAAGTATATT |
| RIM2 | CTGTGTTGAGTAGTCTATAT |
| TPC1 | TCGGATTGTGGAAAAAAGAG |
| YPR011C | AATGATTATGATTGCCAGTA |

**Table S4.** qRT-PCR calculations:

We first calculated the average Cq for each biological replicate (each had 3 technical replicates). Then we calculated the fold change for each biological replicate using the following formula:

$$\frac{2^{-\left(C_{q_{g,c}}-C_{q_{g,e}}\right)}}{2^{-\left(C_{q_{h,c}}-C_{q_{h,e}}\right)}}$$

Where g indicates gene of interest, c indicates crispri plasmid, e indicates empty plasmid, and he indicates housekeeping gene. The fold repression is defined as the reciprocal of the fold change. After that, we took the average of the three biological replicates to get the average fold change, fold repression, and standard deviations listed in the table below.

| | **Average** | **Standard deviation** |
|---|---|---|
| Fold change in expression of YPR011C CRISPRi vs. empty vector | 0.25 | 0.05 |
| Fold change in expression of PIC2 CRISPRi vs. empty vector | 0.20 | 0.02 |
| Fold repression of YPR011C by CRISPRi | 4.14 | 0.75 |
| Fold repression of PIC2 by CRISPRi | 5.04 | 0.47 |

## Supplementary Figures

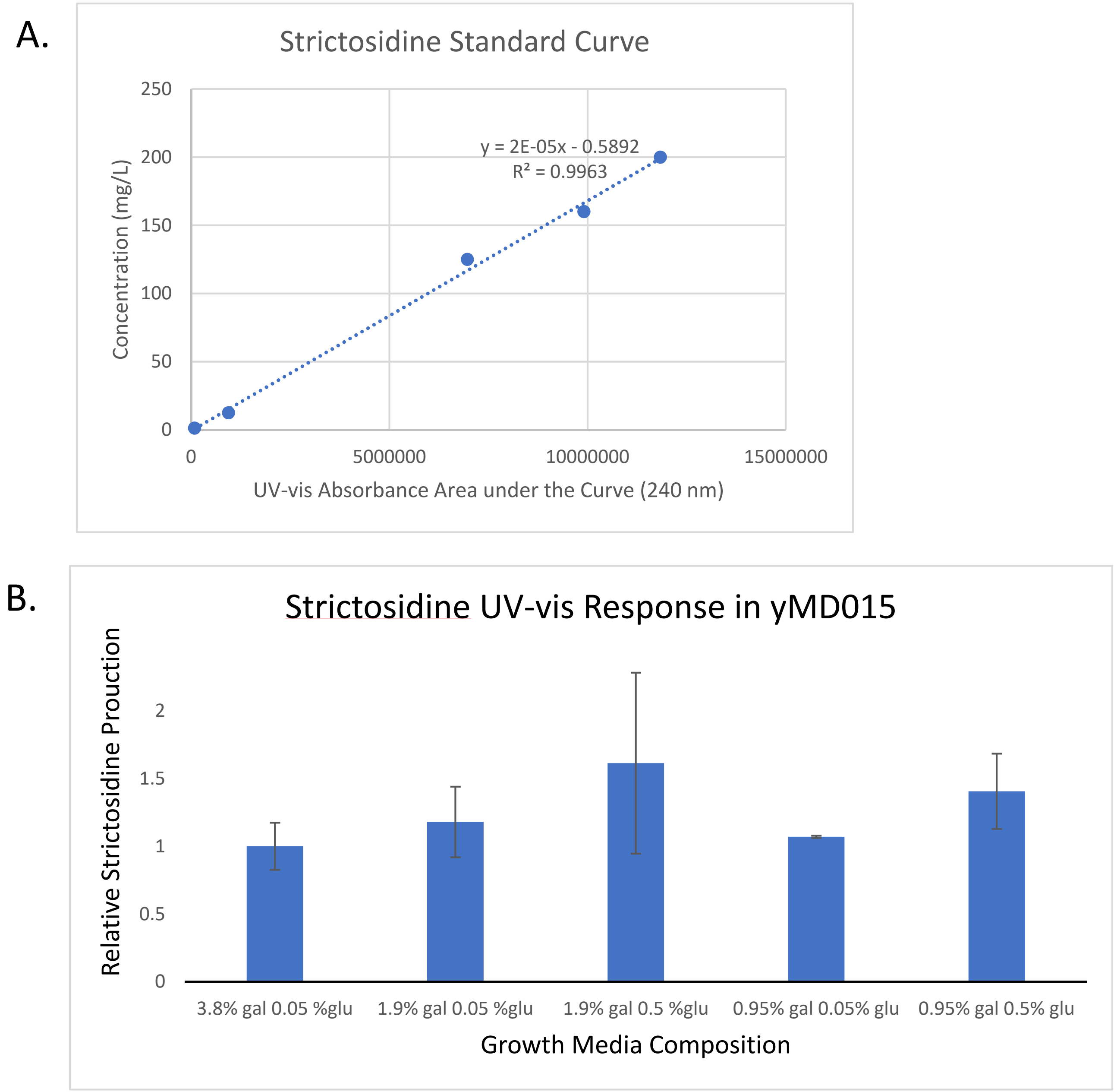


**Figure S1.** Strictosidine standard curve and media optimization. (A) strictosidine standard curve generated from running purified strictosidine on Triple-Quadrupole LCMS. (B) yMD015 was grown in varying concentrations of galactose and glucose rich media, and relative strictosidine titers were measured after 5 days of growth in media, showing that 1.9% galactose and 0.5% glucose was the optimal sugar concentrations out of the variations tested.

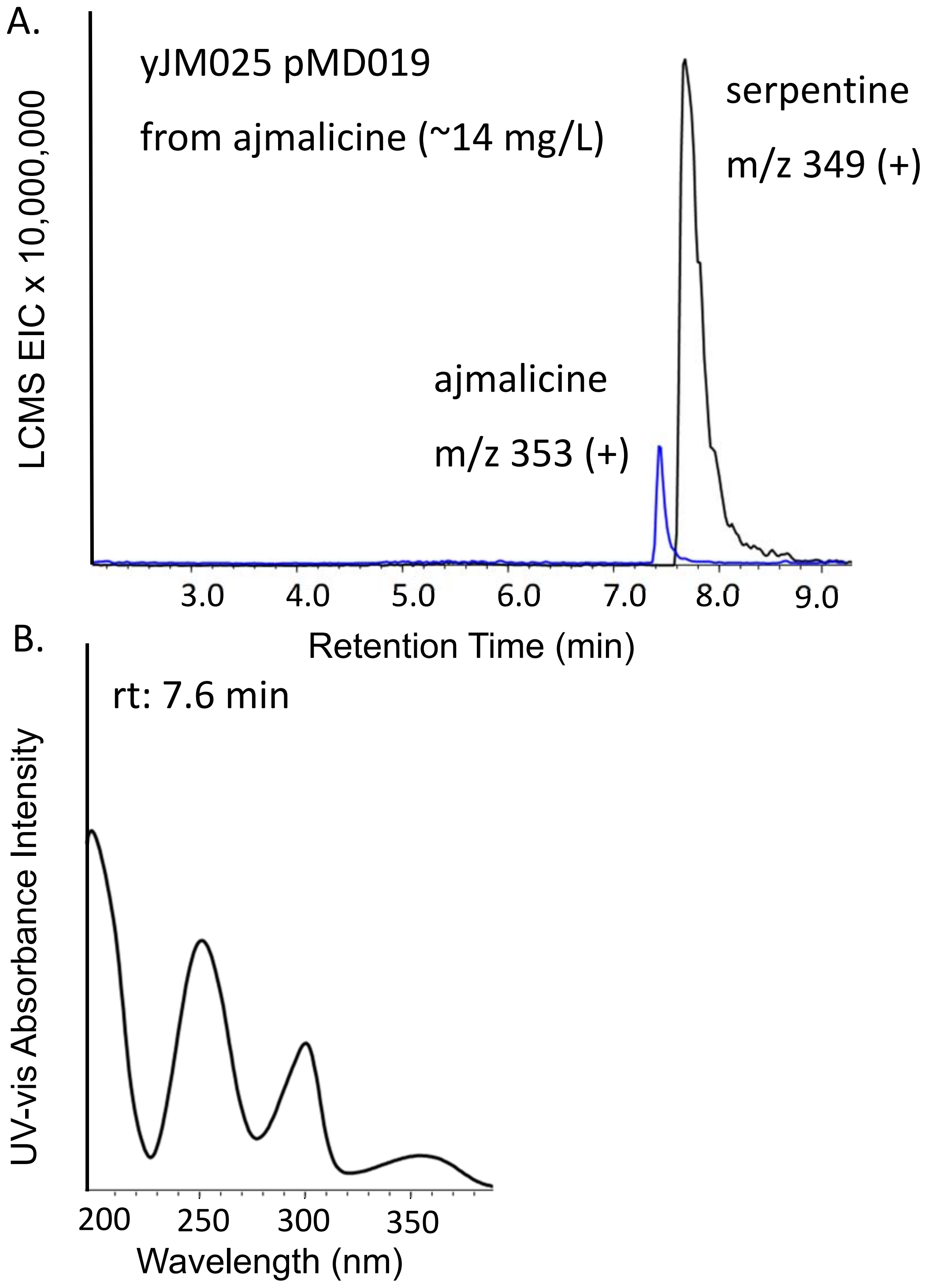


**Figure S2.** Serpentine biosynthesis from ajmalicine (A) LCMS trace of yJM025 pMD019 (Table 1,Table S2) fed with 20 μg ajmalicine and extracted after 24 hrs growth in 1.9 % gal 0.5 % glu media (3 mL cultures), mass filtered for ajmalicine (m/z 353+) and serpentine (m/z 349+) (B) UV-vis absorbance of serpentine (m/z (+) 349, rt 7.6 min)

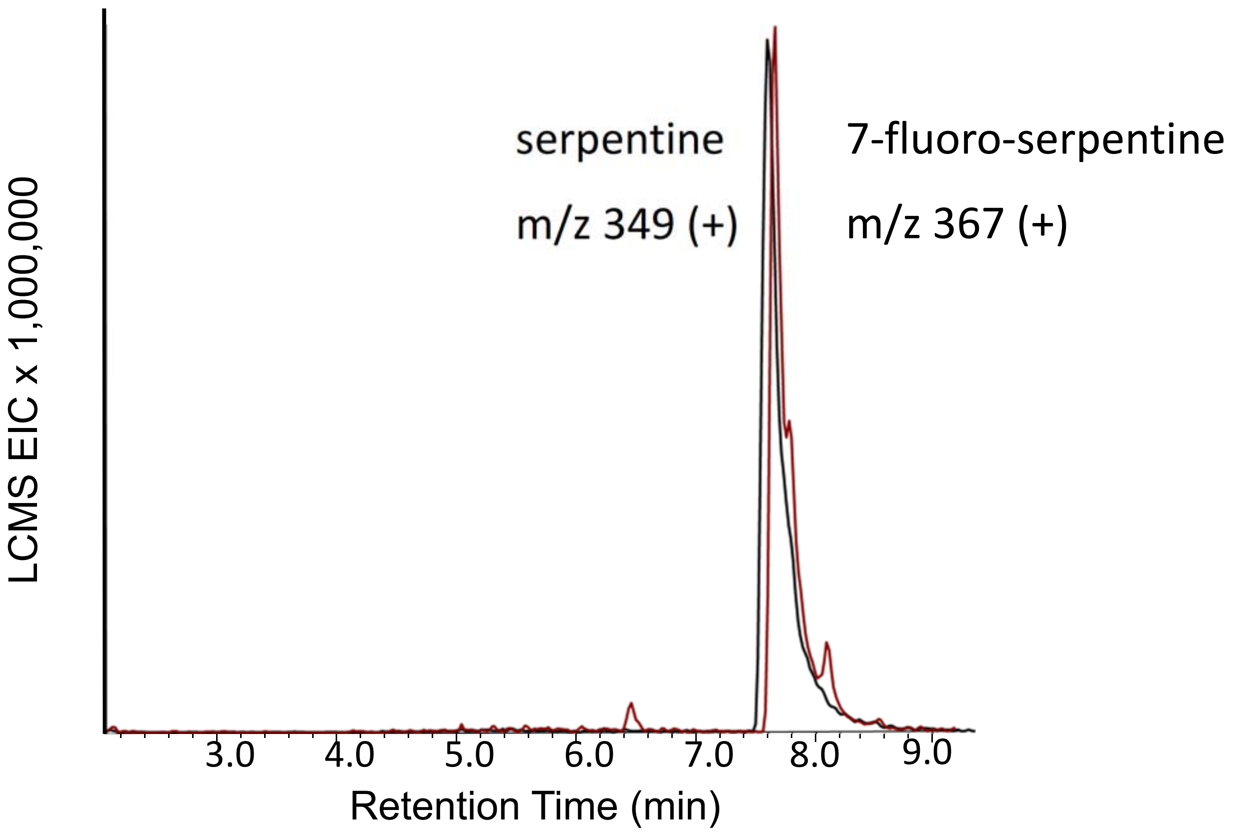


**Figure S3.** *De novo* biosynthesis of 7-fluoro-serpentine. Overlayed LCMS trace of two yMD034 cultures, one fed with tryptamine and one fed with 7-fluoro-tryptamine. after 4 days growth in 1.9 % gal 0.5 % glu media (3 mL cultures), mass filtered for serpentine (m/z 349+) and 7-fluoro-serpentine (m/z 367+). The culture fed tryptamine produced a 349+ peak but not the 367+ peak (black), and the culture fed 7-fluoro-tryptamine produced a 367+ peak, but not the 349+ peak (maroon).

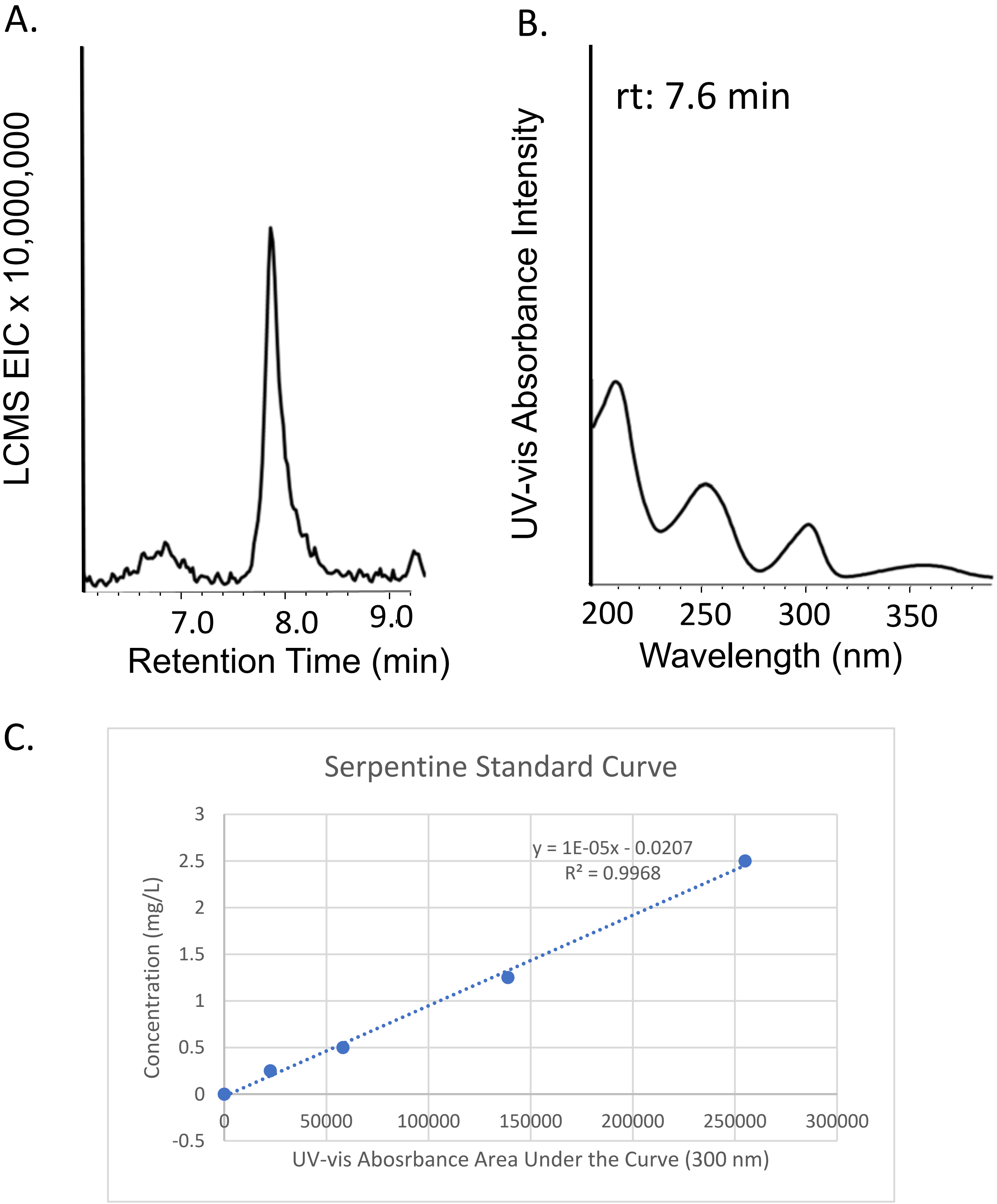


**Figure S4.** Serpentine Hydrogen Tartrate Standard. A) LCMS trace of serpentine hydrogen tartrate standard (m/z 349+) (B) UV-vis absorbance of serpentine (m/z (+) 349, rt 7.6 min) (C) standard curve generated using serpentine standard correlating concentration with UV-vis absorbance area under the curve at 300 nm.

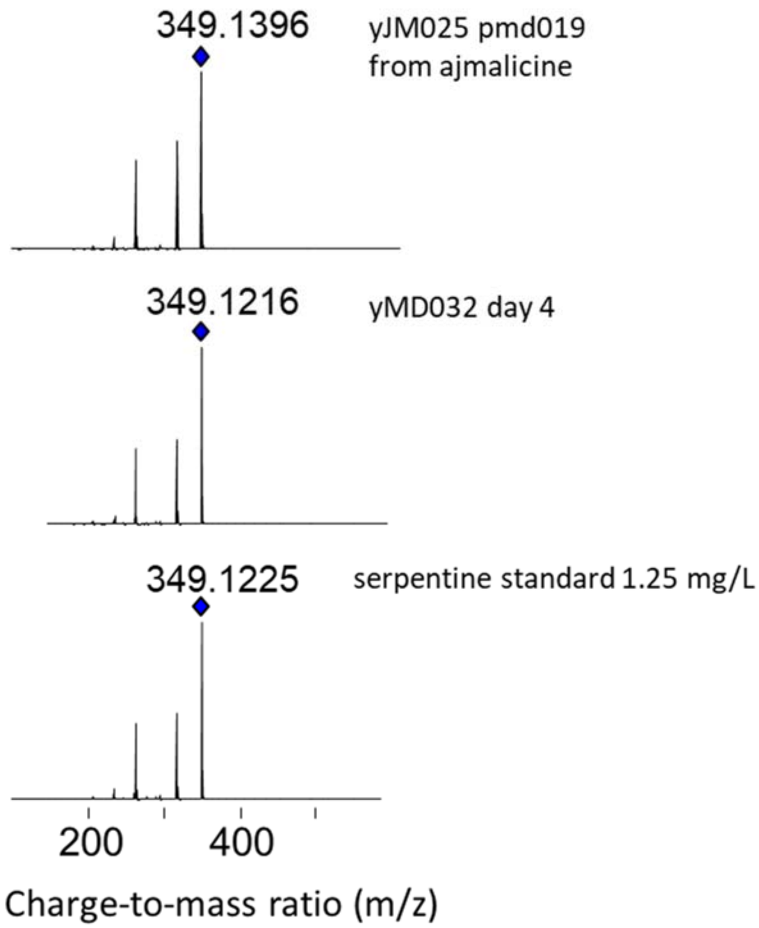


**Figure S5.** *De novo* serpentine MS/MS spectra compared with standard and single biotransformation strain. High-resolution MS/MS data was collected on an Agilent 6545 LCQ-TOF MS with a 25 V collision voltage. MS/MS fragmentation pattern of *de novo* yMD032 349+ compound compared with yJM025 pMD019 fed ajmalicine and serpentine hydrogen tartrate standard, demonstrating evidence of *de novo* serpentine production.

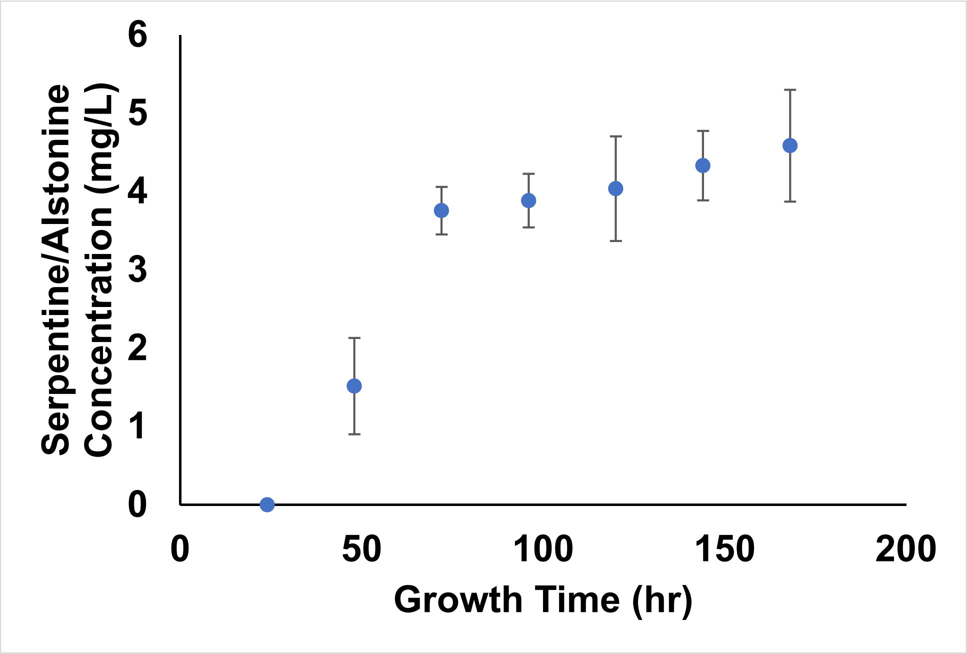


**Figure S6.** Time course of *de novo* Production of Serpentine from yMD032. The strain yMD032 was grown in YP 0.5% glucose 1.9% galactose media over time and extracted in methanol at 24 hr time points then analyzed on LCMS and compared with the standard calibration curve (S4) to quantify titers at each timepoint.

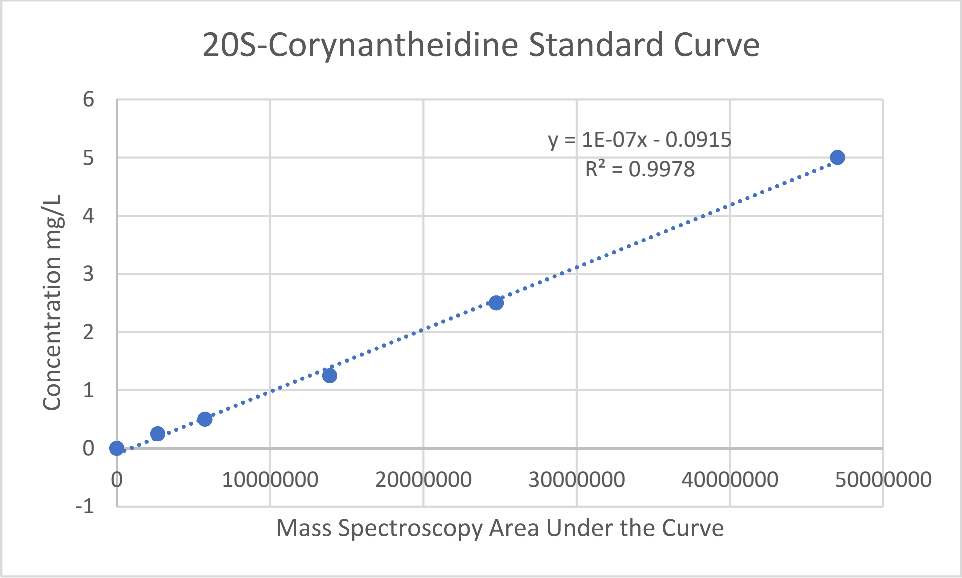


**Figure S7.** (20*S*)**-**Corynantheidine standard curve. Corynantheidine standard curve generated by measuring different concentrations of standard on LCMS where the area under the peak was recorded and plotted against concentration.
